# Autochthonous and Imported Visceral Leishmaniasis in Bulgaria—Clinical Experience and Treatment of Patients

**DOI:** 10.3390/pathogens13030205

**Published:** 2024-02-26

**Authors:** Kamenna Vutova, Nina Yancheva-Petrova, Rossitsa Tchipeva, Valeri Velev

**Affiliations:** 1 Medical College, Medical University of Sofia, 3 “Y. Filaretova St.”, 1606 Sofia, Bulgaria; 2 Medical Center of University Hospital “St. I. Rilski” EAD, 15 “Acad. I. Geshov Blvd.”, 1606 Sofia, Bulgaria; 3 Department of Infectious Diseases, Parasitology and Tropical Medicine, Faculty of Medicine, Medical University of Sofia, 17 “Acad. I. Geshov Blvd.” 17, 1606 Sofia, Bulgaria; n.yancheva-petrova@medfac.mu-sofia.bg (N.Y.-P.); drtchipeva@mail.bg (R.T.); velev_md@abv.bg (V.V.); 4 Specialized Hospital of Infectious and Parasitic diseases “Prof. I. Kirov”, 17 “Acad. I. Geshov Blvd.”, 1606 Sofia, Bulgaria

**Keywords:** autochthonous visceral leishmaniasis, diagnosis, treatment

## Abstract

Visceral leishmaniasis (VL) is a severe endemic disease with a fatal outcome if left untreated. The symptoms of patients are diverse and atypical. Against the background of anemia and pancytopenia, the condition of the patients gradually worsens with marked cachexia. Through sharing our experience, we aim to draw attention to this deadly disease. Clinical and laboratory data for 58 patients with VL treated over a forty-five-year period are presented. The diagnosis was established within a duration of 1 to 28 months of illness. Continuous fever (38–42 °C), splenomegaly, hepatomegaly, severe anemia (decreased hemoglobin to lowest values of 31 g/L), leucopenia (lowest values of leucocytes et 0.5 g/L), and thrombocytopenia (reduced thrombocyte count to 29 g/L) were observed. The diagnosis was made on the basis of microscopic evidence of amastigote forms in bone marrow smears and serological tests. The patients were treated with Glucantime for 17 to 21 days. Relapses were observed in seven patients (12.1%) and fatal outcome was observed in two patients (3.5%) during treatment, who developed acute respiratory and cardiovascular failure. In Bulgaria, Visceral leishmaniasis is primarily endemic in the southern regions and should be suspected not only in patients who have returned from tropical and subtropical countries, but also in those who have not traveled abroad. The challenges associated with VL stem from delayed diagnosis of patients, as this disease remains unrecognized by physicians.

## 1. Introduction

Visceral leishmaniasis is a severe ongoing disease with a fatal outcome in patients without treatment. This disease is mostly distributed in seven countries (Brazil, Ethiopia, India, Kenya, Somalia, South Sudan, and Sudan), where more than 90% of the worldwide VL cases have been reported [1]. An estimated 500,000 new cases of VL and 50,000 deaths occur annually, which are thought to be underestimated [2,3]. In 2022, 12,842 new VL cases (12,773 autochthonous and 69 imported) were reported to the WHO [4]. The Eastern Mediterranean and European regions are also considered endemic areas, although the reported cases are fewer compared to Southeast Asia and Africa. Contrary to other exotic vector-borne diseases, namely Dengue and Chikungunya, which caused health emergencies in Europe in the last decade and thus raised the interest of the scientific community, Visceral leishmaniasis has been neglected, leading to suboptimal knowledge of healthcare professionals about its prevalence, symptoms, and diagnostic options [5,6].

The spread of this disease is naturally endemic, but individual cases and epidemics are also observed. In the European continent, visceral leishmaniasis in animals and/or humans has been reported in 22 countries including Albania, Austria, Bosnia and Herzegovina, Bulgaria, Croatia, Cyprus, France, Germany, Greece, Hungary, Italy, Kosovo, Malta, Montenegro, North Macedonia, Portugal, Romania, San Marino, Serbia, Slovenia, Spain, and Ukraine, with only 17 countries reporting autochthonous human leishmaniasis cases [7].

Imported case of Visceral leishmaniasis in an adult infected in Greece was first presented in Bulgaria in 1921 [8]. Primarily children infected in Greece and Macedonia, as individual cases been published [9,10]. However, in 1952, autochthonous cases of VL were also reported, involving 31 children and 3 adults from settlements near the Greek and Turkish borders. [11]. For more than a quarter of a century, there have been no publications describing patients with Visceral leishmaniasis, but it is likely that this disease was the cause of deaths of unknown etiology. In 1989, six patients with Visceral leishmaniasis (two autochthonous, three infected in Greece, and one in Iraq), hospitalized and treated in the Clinic for Tropical and Parasitic Diseases in Sofia between 1976 and 1989, were reported, drawing attention to this “forgotten” disease [12,13]. From 1998, there was an increase in autochthonous cases of Visceral leishmaniasis among Bulgarian citizens across the country, characterized by a severe clinical course [14,15,16,17]. For 25 years (1988–2012), a total of 122 cases were registered, 118 of which were autochthonous from southern regions and four were imported [18]. 

The clinical course of Visceral leishmaniasis is nonspecific for months and even years, contributing to the delayed diagnosis of infected patients and, subsequently, fatal outcomes. For a long time, they were treated on an outpatient basis and were hospitalized several times, without establishing the cause of the fever and worsening of their condition. The increasing frequency of severe cases and the lack of recognition of this disease are the reasons for presenting clinical data from cases observed by us over a 45-year period. We expect that our experience will be useful to medical doctors in European countries, where this disease is uncommon, as well as in response to the increasing number of emigrants from endemic regions worldwide.

## 2. Materials and Methods

Fifty-eight patients with Visceral leishmaniasis were observed, examined, and treated from 1976 to 2021 at the Department of Infectious Diseases, Parasitology and Tropical Medicine in Sofia. The age of the patients varied from 1 to 76 years (mean age 37 ± 12.6), of which 43 (74.1%) were men and 15 (26%) were women. A total of 7 of the patients (12.1%) were children and 51 (87.9%) were adults (Table 1). Most individuals involved in the study were over 21 years old (48, 82.8%), followed by individuals between 11 and 20 years old (4, 7%) and between 1 and 10 years old (5, 8.6%).

Eleven cases were imported: one from Greece, one from Iraq, and seven Bulgarians who fell ill during their stay in Greece, as well as two from South Italy. The other 47 patients had no history of traveling abroad. The autochthonous VL cases were predominantly from the southwest region of Bulgaria—31 patients (66%), 13 cases (27.7%) were from Central South Bulgaria, and only 3 patients (5.2%) were from Northern Bulgaria. The largest number of patients with VL are from the town of Petrich and nearby villages. The patients are mainly men who report a longer stay in nature—fishing, working near rivers, lakes, or in the forest. Nearly half of the patients deny having traveled outside the city.

The diagnosis of Visceral leishmaniasis was established based on clinical symptoms, hematological tests (complete blood count, biochemical blood tests), and microscopic examination of bone marrow to demonstrate amastigote forms of *Leishmania* sp. after Giemsa staining. Diagnostic microscopic methods for protozoa such as *Leishmania* sp. are with a high magnification of 1000× with an objective lens of 40× and 10× ocular with immersion oil.

Serological methods, such as the indirect immunofluorescence assay test (IFAT), enzyme-linked immunosorbent assay (ELISA), and polymerase chain reaction (PCR) of the punctate were carried out at the Bulgarian National Center of Infectious and Parasitic Diseases in Sofia. The samples were examined as part of the routine diagnostic process in each patient.

Patients with VL were followed up after 1, 6, 12 months, and more in cases of recurrence with clinical examination, laboratory tests (hematological tests and ELISA IgG test for VL), and microscopic examination of bone marrow to demonstrate amastigote forms of *Leishmania* sp. after Giemsa staining.

For the purposes of this study, we used data from the disease histories of patients and our own observations. All adult patients provided written informed consent at the time of the clinical examination and a parent or a child’s guardian provided written informed consent on their behalf. Patient records and information were anonymized and de-identified before analysis. According to the Bulgarian legislation, Visceral leishmaniasis has become a mandatorily notifiable disease since 1978 [19]. Notification and registration of all cases of Visceral leishmaniasis have been regulated in two ordinances [20,21].

## 3. Results

### 3.1. Clinical Observations of Infected Patients with VL

The patients with Visceral leishmaniasis were mainly from the southern regions and three were from Northern Bulgaria. The duration of the disease course ranged from 10 days to 28 months. A total of 21 (36.2%) patients were diagnosed in the first month, 6 patients (10.3%) within 2 months of illness, 4 patients (6.9%) within 3 months, 7 patients (12.1%) after 4 months, 8 patients (13.7%) after 6 months, 3 patients (5.2%) after 8 months, 3 patients (5.2%) after 12 months, and 1 patient at 5, 7, 10, 11, 18, and 28 months since the onset of the disease, respectively. These results show that the period of diagnosing patients with VL is long. The diagnostic process in 63.8% of them lasts more than one month. 

The incubation period in observed patients is difficult to determine due to the fact that it varies from 20 days to 18 or more months. Humans become infected during the summer months, when the factors for the spread of the invasion are present. These factors include the insects of the phlebotomus genus and the reservoirs—dogs, jackals, foxes, rodents, and a favorable environmental temperature. The disease manifests in the autumn–winter period, and there are only a few patients in the spring of the following year.

The initial diagnoses of patients with Visceral leishmaniasis are colds, viral diseases, influenza, pneumonia, chronic myelofibrosis, a lymphoproliferative process, lymphoma, non-Hodgkin lymphoma, viral hepatitis, sepsis, infectious mononucleosis, rickettsiosis, malaria, and others. With these diagnoses, the patients were treated several times (weeks, months) with antipyretic drugs and antibiotics without affecting the high temperature and were hospitalized more than once without improvement. Twenty-three (39.6%) were hospitalized once, 16 (27.6%) twice, 8 (13.8%) three times, 4 (7%) four times, and 1 (1.7%) 7 times before specifying the diagnosis. These data indicate that a significant part of the patients (60.4%) with visceral leishmaniasis have two or more hospitalizations before diagnosis. 

All patients with VL reported high temperatures over 38 °C, severe fatigue, muscle weakness, significant weight loss from 5 to 39 kg., and loss of appetite. Against the background of fever, one of the patients with a one-month illness reported severe pain in the left lower rib. He had an enlarged spleen 10 cm below the costal arch. 

In all patients, the disease manifested with prolonged fever lasting from months to more than a year. The temperature remained in the range of 38 to 42 °C and is characterized by 1 to 3 peaks per day, accompanied by shaking and not affected by antipyretics. The temperature ranged up to 38 °C in 6 of the patients (10.3%), up to 38.1–39 °C in 25 (43.1%), up to 39.1–40 °C in 23 (39.7%), up to 40.1–41 °C in 3 (5.2%), and 42 °C in 1 (1.7%) patient. These results prove that a significant part of the patients (89.7%) with VL had very high temperatures of over 39 °C (Table 1).

**Figure 1:**
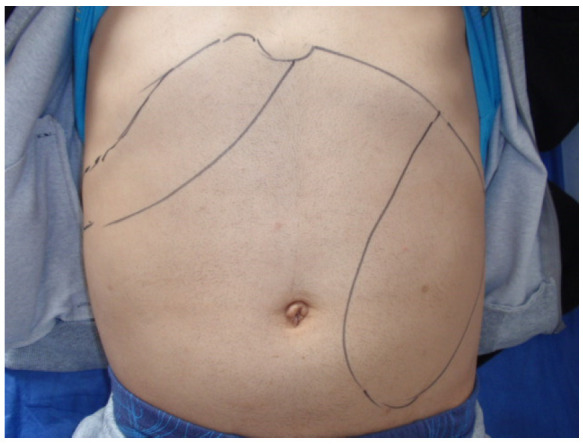
Splenomegaly and hepatomegaly in a 35-year-old patient (Ch.M. 2013) with recurrent VL and hepatitis B.

**Figure 2:**
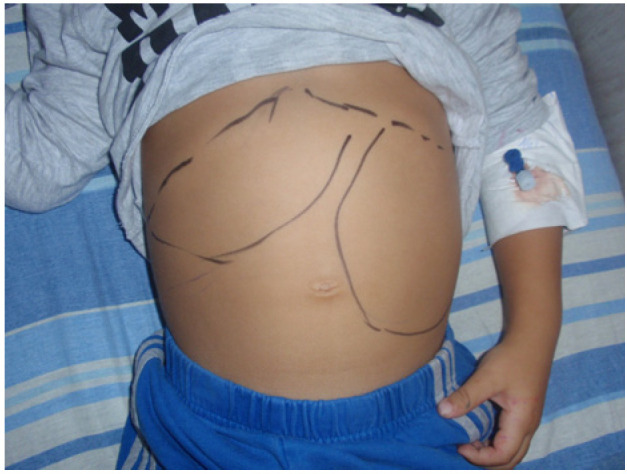
Splenomegaly and hepatomegaly in a 3-year-old child (G.S. 2014) with VL two weeks after onset of illness.

All patients had hepatomegaly and splenomegaly, except for one splenectomized patient. An enlarged liver was palpated at 1–3 cm in thirty-four of the patients (58.6%), at 4–6 cm in sixteen (27.6%), at 8 cm in four (6.9%), and single cases were at 9 and 12 cm below the costal arch. An enlarged spleen was palpated with huge dimensions at 10 to 20 cm (Figure 1 and Figure 2) in twelve patients (20.7%), at 7–9 cm in five (8.6%), at 4–6 cm in twenty-eight (48.3%), and 1–3 cm in thirteen (22.4%). These observations provide information about a significant spleen enlargement more than 4 cm below the costal arch in 77.6% of patients with Visceral leishmaniasis. The liver was enlarged more than 4 cm below the costal arch in a smaller part (in 37.9%) of the observed patients with Visceral leishmaniasis.

With the progression of the disease, thrombocytopenia leads to hemorrhagic complications, such as spontaneous bleeding from nasal and oral mucosa. In six (10.3%) of the patients, epistaxis was observed, hemoptysis in two (3.5%), and melena in one (1.7%). 

Patients with Visceral leishmaniasis have a pronounced paleness of the skin, especially marked on the face, which is defined as porcelain skin (20.7%), grayish color (26%), and a dark gray earthy tinge (5.2%). Enlarged lymph nodes were found in eighteen (31%) of patients with VL. Four of the patients had edema on their legs and one on the whole body.

The significant delay in determining the etiological diagnosis (6–12 and more months) and the long-term treatment with corticosteroids, cytostatics, and blood transfusions are the cause of the impaired condition of the patients with VL.

### 3.2. Laboratory Evaluation of Infected with VL Patients

Hematological abnormalities (anemia, leukopenia, thrombocytopenia) are highly pronounced in patients with VL (Table 1). Anemia was observed in all of them—reduced values of hemoglobin (Hb) up to 31 g/L (reference value 120–130 g/L) and erythrocytes count (Er) up to 1.7 g/L. Hemoglobin values are very low: 31 g/L in one patient (1.7%), from 41 to 50 g/L in two (3.4%), from 51 to 60 g/L in seven patients (12.1%), from 61–70 g/L in another seven patients (12.1%), in six (10.3%) from 71 to 80 g/L, in twelve (20.7%) from 81 to 90 g/L, in thirteen (22.4%) between 91 and 100 g/L, in three (5.2%) between 101 and 110 g/L, in three (5.2%) between 111 and 120 g/L, and more than 121 to 130 g/L in four (6.9%). These results indicate that a significant part of the patients with VL has heavy anemia when hemoglobin is less than 65 g/L (20.7%), expressed anemia when hemoglobin levels vary between 65 and 79 g/L (17.2%), moderate anemia with hemoglobin at 80–94 g/L (43.1%), and mild anemia with hemoglobin at 95–110 g/L (10.3%). 

Leukopenia was found nearly in all patients, which is a low leukocyte count of up to 0.4 g/L (reference value 3.5–10.5 g/L). The largest relative share was patients with leukocytes in the range from 1 to 2 g/L in twenty-three (39.7%), followed by a group with leucocytes within the range of 2.1–3.0 g/L in eighteen (31%), and between 3.1 and 3.4 g/L in eleven patients (19%) with Visceral leishmaniasis. Marked leukopenia from 0.4 to 0.9 g/L was observed in four patients (6.9%). Leucocyte counts were normal in two (3.4%) of the patients within the range of 4.1 to 5 g/L. These results show that almost half of the patients (46.6%) with Visceral leishmaniasis had expressed leukopenia (0.4–2 g/L).

Thrombocytopenia was found in forty-eight (82.8%) of the patients up to the lowest platelet count of 12 g/L (reference value 130–440 g/L). Exceptionally low platelet counts up to 30 g/L were found in seven patients (12.1%) and within the range from 61 to 70 g/L in six patients (10.3%). There were five patients (8.6%) in each of the following groups from 41 to 50 g/L, 51 to 60 g/L, 71–80 g/L, 91–100 g/L, and 101–110 g/L. Four patients (6.9%) had platelet counts higher than 81 to 90 g/L, three patients (5.2%) had a platelet count between 111 and 120 g/L, and three patients (5.2%) were within the limits between 131 and 140 g/L. Platelet counts were normal in ten of the patients (17.2%). These results demonstrate that half of the patients (48.3%) with Visceral leishmaniasis have very low thrombocyte count (0.4 to 80 g/L).

A significant part of patients, twenty-four (41.4%), with VL have a high erythrocyte sedimentation rate of 100–180 mm per hour (reference values of 15 mm/h for men and 20 mm/h for women).

Marked hypoalbuminemia (albumin up to 20 g/L) was observed in five patients (8.6%), with the lowest values being 14 g/L. In 22 patients (37.9%), the serum albumin ranged from 21 to 34 g/L with the reference value being 35–52 g/L. With these results, it was found that 46.6% of the observed patients had low serum albumin (Table 1).

### 3.3. Etiological Diagnosis of Infected Patients with VL

**Figure 3:**
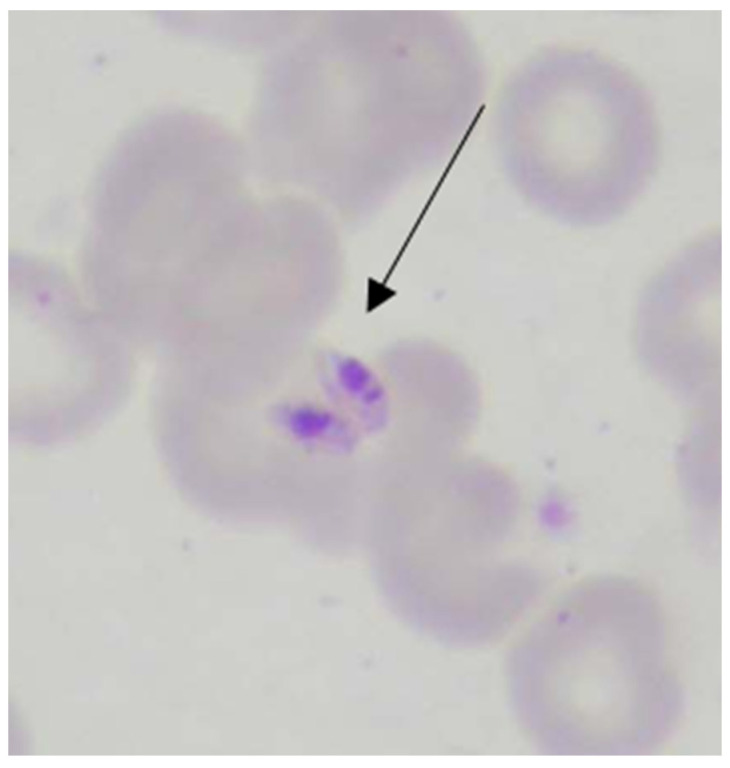
Amastigotes of *Leishmania* sp. (1000× magnification) in bone marrow aspirate extracellularly (Giemsa staining) in a patient (D.D 2004).

**Figure 4:**
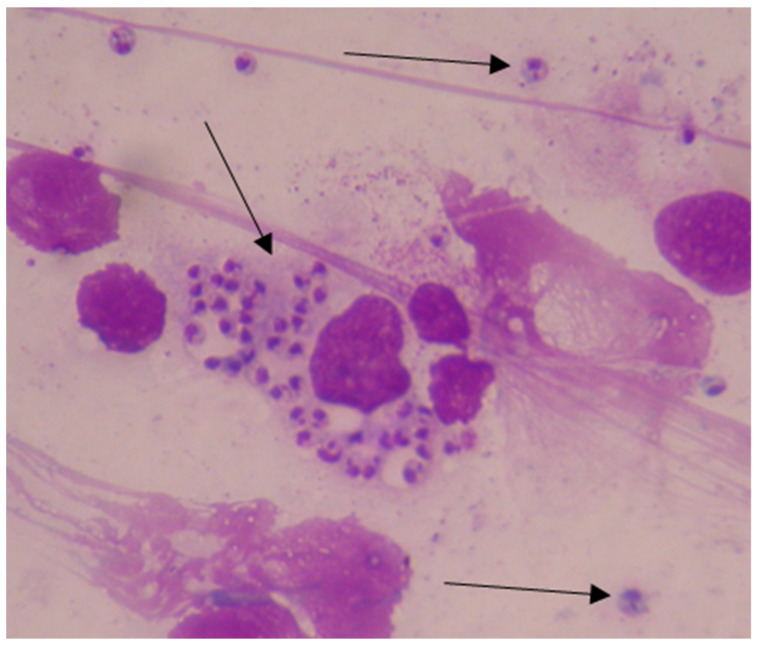
Amastigotes of *Leishmania* sp. (1000× magnification) in bone marrow (Giemsa staining) intracellularly and extracellularly in a patient (I.Y 2010).

The diagnosis of Visceral leishmaniasis was made on the basis of microscopic evidence of amastigote forms in bone marrow smears (Figure 3 and Figure 4). Fifty-six of the patients were tested by Giemsa staining smears, and in seven of them (12.5%) the result was positive in the bone marrow when taking a second sample and in two patients (3.5%) in the third biopsy. In two patients (3.5%), *Leishmania* sp. amastigotes were found in a liver biopsy. Out of all 56 patients, in whom the bone marrow was examined, in 47 (83.9%), the diagnosis was established with the first puncture.

In two patients with Visceral leishmaniais, morphological examination of Giemsa-stained samples was negative. However, the diagnosis was confirmed through positive ELISA and PCR tests, the presence of clinical symptoms, and improvement observed after initiating treatment with Glucantime. The results of the serological tests show that the ELISA test is positive (>1.1) in 95% of the examined patients with VL and it can be used in cases with an inconclusive positive result from the bone marrow test. The results with IFAT were positive (>1:80) in six of the eleven examined patients (54.6%), and, in two patients (18.2%), the positive results appeared upon the second examination. Out of the 39 patients tested by ELISA, the results were negative in two cases (5.1%). In two patients, positive ELISA results were reported after repeat testing. In a patient where no microscopic amastigotes were found in the bone marrow, the PCR of the punctate was positive.

### 3.4. Treatment of Infected Patients with VL

All patients with Visceral leishmaniasis were treated with Meglumine antimoniate (Glucantime) at a dose of 20 mg per kg body weight per day, i.m. twice daily for 17 and 21 days. The course of treatment in 6 patients was shorter than 21 days due to the occurrence of side effects, such as changes in the electrocardiographic examination and due to the insistence of the people to be discharged. Recurrence was noted in 4 of these patients.

After starting the etiological treatment, the temperature of the patients decreased and normalized after the fourth day; the condition improved and a tendency to increase hemoglobin, leukocytes, and platelets was noted. Relapses were reported in seven patients (12.1%) after treatment with Glucantime, potentially attributed to inadequate treatment duration (15 and 17 days) and the presence of concomitant diseases, such as viral hepatitis B and HIV infection. Two of the patients were treated due to relapse with liposomal Amphotericin B (AmBisome) at a dose of 3 mg per kg of body weight per day, i.v. on the 1st–5th, 10th, 14th, and 21st day. One patient was treated for relapse with Mltefosine (Impavido) capsules 50 mg, administered for 28 days, at a dose of 2.5 mg per kg body weight per day, up to 150 mg per day.

From all 58 patients with Visceral leishmaniasis, a fatal outcome during treatment was observed in 2 hospitalized patients (5.1%), who developed acute respiratory and cardiovascular failure. These patients had a long duration of febrility (6 and 12 months), and were treated for months with antibiotics, corticosteroids, cytostatics, and blood transfusions. For six of the patients, whose state improved following treatment with Glucantime, and who were discharged from the hospital, we have data of a fatal outcome one and two years later but due to the complications of chronic hepatitis B in three patients, and a malignancy in two patients. One patient with an HIV infection died regardless of repeated treatment with Glucantime and liposomal Amphotericin B. He had an HIV infection history of more than 10 years. Despite achieving optimal viral suppression for HIV, the patient had a suboptimal immunological response to antiretroviral therapy and severe immunodeficiency.

## 4. Discussion

The natural outbreaks of Visceral leishmaniasis in Bulgaria, established 60 years ago, have persisted to the present day. The data indicating the high number of registered cases in the two regions suggest the ongoing transmission of VL in Bulgaria [18]. In investigating the potential role of dogs as carriers in urban areas, studies were conducted in the Southwestern Bulgarian city of Petrich, revealing domestic dogs infected with Visceral leishmaniasis [22,23]. The data on 25 patients from the town of Petrich and 4 patients from surrounding villages diagnosed and treated by us support the possibility of the presence of natural outbreaks of leishmaniasis. This fact gives reason to direct the attention of doctors and veterinarians to suspect the disease VL, and also to take prophylactic measures regarding the reservoirs and carriers of *Leishmania* sp.

Visceral leishmaniasis is severe in patients with a long duration of illness, with accompanying diseases, and is more severe and often fatal in those infected with HIV infection [24,25,26,27]. The significant delay in establishing the etiological diagnosis in the observed patients (duration of illness of six or more months), as well as the long-term treatment with corticosteroids and cytostatics, are the cause of the impaired general condition of the patient. In patients experiencing fever over 39 °C, lack of improvement following antibiotic treatment, and the presence of splenomegaly, anemia, leukopenia, and thrombocytopenia, it is advisable to consider ruling out Visceral leishmaniasis, even in the absence of a history of travel to tropical or subtropical countries.

According to our observations, the microscopic examination of Giemsa-stained bone marrow aspirate with evidence of amastigote forms is the most reliable for the etiological diagnosis. However, in some patients (16.1%), *Leishmania* parasites were detected through repeated bone marrow puncture. In these cases, serological methods are required without being able to confirm or reject the diagnosis with certainty. Both methods are necessary in cases with fever of unknown origin.

Treatment with Glucantime has shown success with the first therapeutic course in the majority of patients (79.3%), but to achieve a cure, according to our experience, a minimum duration of 21 days is necessary. The World Health Organization also provides guidelines suggesting treatment adjustments tailored to each country and patient, ranging from 20 to 28 days [28]. This underscores the need for an individualized approach to each patient, considering the duration of treatment and the potential for repeated courses. In Bulgaria, persons with VL are subject to dispensary observation for up to one year. Regular control examinations and follow-up for up to 12 months or longer, if necessary, are crucial.

## 5. Conclusions

The clinical course of VL is nonspecific for months and years in many cases, contributing to the delayed diagnosis of infected patients and, subsequently, fatal outcomes. For a long time, they were treated and hospitalized several times without establishing the cause of the fever and worsening of their condition. In Bulgaria, VL is endemic and should be suspected both in patients who returned from tropical and subtropical countries, as well as in those without information of prior travels. The problems related to this disease are due to the late diagnosis of the patients.

The increasing frequency of severe cases and the lack of recognition of this disease are the reasons for presenting clinical data from cases observed in our hospital and to draw attention to this “forgotten” disease in the country. We expect that our experience will be useful to medical doctors in European countries, where this disease is uncommon, as well as in response to the increasing number of emigrants from endemic regions worldwide.

## Figures and Tables

**Table 1:** Data of patient with VL—age, sex, laboratory results.

Data of Patients with VL	Number of Cases	Relative Share
Male	43	74.1%
Female	15	26.%
Children	7	12.1%
Adults	51	87.9%
Imported VL cases	11	19%
Autochthonous VL cases	47	81%
Relapses	7	12.1%
Fatal outcome	8	10.3%
During treatment	2	3.5%
Dehospitalized patients	6	8.6%
High temperature over 39 °C	52	89.7%
38 °C	6	10.3%
38.1–39 °C	25	43.1%
39.1–40 °C	23	39.7%
40.1–41 °C	3	5.2%
42 °C	1	1.7%
Hepatomegaly	58	100%
Splenomegaly	57	98.3%
Anemia	58	100%
Mild anemia (hemoglobin 95–110 g/L)	6	10.3%
Moderate anemia (hemoglobin 80–94 g/L)	25	43.1%
Expressed anemia (hemoglobin 65–79 g/L)	10	17.2%
Heavy anemia (hemoglobin less than 65 g/L)	12	20.7%
Leucopenia	58	100%
Expressed leukopenia (0.4–2 g/L)	27	46.6%
Thrombocytopenia	48	82.8%
Very low thrombocyte count (0.4 to 80 g/L)	28	48.3%
High erythrocyte sedimentation rate (100–180 mm/h)	24	41.4%
Hypoalbuminemia	27	46.6%
Marked hypoalbuminemia (albumin up to 20 g/L)	5	8.6%

## Data Availability

The data presented in this study are available on request from the corresponding author.
